# High Prevalence of *Mycoplasma pneumoniae* and *Chlamydia pneumoniae* in Children with Acute Respiratory Infections from Lima, Peru

**DOI:** 10.1371/journal.pone.0170787

**Published:** 2017-01-27

**Authors:** Juana del Valle-Mendoza, Fiorella Orellana-Peralta, Alvaro Marcelo-Rodríguez, Eduardo Verne, Mónica Esquivel-Vizcarra, Wilmer Silva-Caso, Miguel Angel Aguilar-Luis, Pablo Weilg, Verónica Casabona-Oré, Claudia Ugarte, Luis J. del Valle

**Affiliations:** 1 School of Medicine, Research and Innovation Centre of the Faculty of Health Sciences. Universidad Peruana de Ciencias Aplicadas-UPC, Lima, Peru; 2 Instituto de Investigación Nutricional, Lima, Peru; 3 Universidad Nacional Federico Villarreal, Lima, Peru; 4 Hospital Nacional Cayetano Heredia, Lima, Peru; 5 Centre de Biotecnologia Molecular (CEBIM), Departament d’Enginyeria Química, ETSEIB, Universitat Politècnica de Catalunya (UPC) Barcelona Tech, Barcelona, Spain; Beijing Institute of Microbiology and Epidemiology, CHINA

## Abstract

**Background:**

*Mycoplasma pneumoniae* and *Chlamydia pneumoniae* are atypical pathogens responsible for pneumonia and a leading cause of morbidity and mortality in low income countries. The study objective is to determine the prevalence of this pathogens in Peruvian children with acute respiratory infections.

**Methods:**

A consecutive cross-sectional study was conducted in Lima, Peru from May 2009 to September 2010. A total of 675 children admitted with clinical diagnoses of acute respiratory infections were tested for *Mycoplasma pneumoniae* and *Chlamydia pneumoniae* detection by polymerase chain reaction (PCR), and clinical symptoms were registered by the attending physician.

**Results:**

*Mycoplasma pneumonia* was detected in 25.19% (170/675) of nasopharyngeal samples and *Chlamydia pneumonia* in 10.52% (71/675). The most common symptoms in patients with these atypical pathogens were rhinorrhea, cough and fever. A higher prevalence of *Mycoplasma pneumoniae* cases were registered in summer, between December 2009 and March 2010.

**Conclusions:**

*Mycoplasma pneumoniae* and *Chlamydia pneumonia* are a significant cause of morbidity in Peruvian children with acute respiratory infections (ARI). Further studies should evaluate the use of reliable techniques such as PCR in Peru in order to avoid underdiagnoses of these atypical pathogens.

## Introduction

Acute respiratory infections (ARI) are a leading cause of morbidity and mortality in children [1,2]. However, the precise epidemiology about etiological agents of ARI in developing countries remains poorly defined [3,4]. Atypical pathogens such as *Mycoplasma pneumoniae* and *Chlamydophila pneumoniae*, are increasingly recognized as important causes of community acquired pneumonia (CAP) worldwide [5].

Both atypical pathogens, *Mycoplasma pneumoniae* and *Chlamydophila pneumoniae* (*Chlamydia pneumoniae*), can cause mild, moderate, or severe acute respiratory infections [6]. Mycoplasma clinical manifestations range from mild cases of tracheobronchitis to severe atypical pneumonia and can be followed by a broad spectrum of extra pulmonary complications [7]. *Chlamydia pneumoniae* is another agent associated with CAP that can also be involved in pharyngitis, bronchitis, and sinusitis [8].

A study in 2016, conducted in children with community acquired pneumonia compared the clinical presentation of typical vs atypical bacterias, and reported that in contrast to *Streptococcus pneumoniae* presentation were fever, cough, rhinitis, and fine crepitation were commonly reported; for both the *M*. *pneumoniae* and *C*. *pneumoniae* the presentation was heterogenous and diminished air entry, ronchi, grunting and rapid breathing were reported [9].

It is difficult to clinically distinguish *M*. *pneumoniae* from *C*. *pneumoniae* infections and hence laboratory tests are essential for the identification of these pathogens [6,10]. Serological detections, although commonly used, are complicated by false negative results in the early acute phase of infection, and the difficulty in obtaining convalescent serum during hospital stays of one week or less. Polymerase chain reaction (PCR) has provided an alternative diagnostic method for etiological agents that are difficult to culture or detect. PCR has been found to be a highly sensitive and specific diagnostic technique for the diagnosis of acute *M*. *pneumoniae* and *C*. *pnemoniae* infections and avoid the risk of false negative results in conventional culture methods [10–12].

The prevalence rate of childhood ARI due to these pathogens is very variable from one country to another due to differences in seasons and geographic areas [6]. In Peru, ARI are a major health problem and more than 300 000 episodes of ARI are reported every year with more than 2700 deaths per year in children under 5 years old [13]. However, despite the increasingly interest of *M*. *pneumoniae* and *C*. *pnemoniae* in many countries, their prevalence in Peru has not been well documented due to the lack of a National surveillance program for these pathogens.

One study in 2008, reported an increase of atypical pneumonia in children from Lima, Peru. In this study, hospitalized children between 1 month to 14 years old were studied for serological detection of atypical pathogens and *Chlamydia pneumoniae* was observed in 17.1% of patients, followed by *Mycoplasma pneumoniae* in 7.1% [14].

There is little information about the role of atypical pathogens in Peruvian children. Our purpose was to use PCR for detection and identification of *M*. *pneumoniae* and *C*. *pneumoniae* in patients under 18 years old with ARI, and to investigate the relationship between their epidemiology and seasonality in Lima, Peru. In addition, we compared the clinical characteristics in patients with *M*. *pneumoniae* and *C*. *pneumoniae* positive samples.

## Materials and Methods

### Patients

Patients under the 18 years hospitalized at the *Hospital Nacional Cayetano Heredia in Lima*—Peru with the diagnosis of acute respiratory infection (ARI) from May 2009 to September 2010 were included in the study. A standard format elaborated by the principal investigator with clinical and epidemiological features was included for: age, sex, symptoms (onset, fever higher than 38°C, cough, headache, arthromyalgia, odynophagia, among others) and clinical diagnosis. All data was collected by the physicians after the patients were diagnosed with ARI in their first day of hospitalization but before they received any antibiotic treatment.

This study was approved by two independent ethics committees: The Research Ethics Board of the Hospital Nacional Cayetano Heredia and Instituto de Investigación Nutricional (IIN). An informed consent was signed by parents or children’s caregivers before enrollment.

### Samples

Nasopharyngeal samples were obtained by inserting a swab into both nostril parallel to the palate (Mini-Tip Culture Direct, Becton-Dickinson Microbiology System, MD 21152, USA) and a second swab from the posterior pharyngeal and tonsillar areas (Viral Culturette, Becton-Dickinson Microbiology Systems, MD, USA). All swabs were placed into tubes containing viral transport medium (minimal essential medium with 2% fetal bovine serum, penicillin and streptomycin 100U/ml, amphotericin B 20 μg/ml, neomycin 40 μg/ml, NaHCO3 buffer). And then the samples were then stored at -4°C until being sent to the Molecular biology laboratory at *Instituto de Investigación Nutricional* (IIN)—*Universidad Peruana de Ciencias Aplicadas* (UPC). Once the samples arrived to laboratory, swabs were discarded and tubes were centrifuged to pellet the cells, which were resuspended in 1.5 ml of PBS (Phosphate Buffered Saline). Two aliquots of each fresh specimen were stored at -20°C to be used for PCR analysis for atypical pathogens.

### Polymerase chain reaction (PCR) for the analysis of atypical pathogens

For the PCR, DNA was extracted from swabs using a QIAamp DNA mini kit (Qiagen, Mississauga, Ontario). The swab was placed in a 1.5 mL microcentrifuge tube containing 300 μL of saline and incubated at 37°C for 10 min on a shaker. Approximately 200 μL of saline then was removed and placed in a fresh 1.5 mL tube, and DNA was extracted according to the manufacturer’s protocol. Polymerase chain reaction was performed with 5 μL of template DNA, polymerase (GoTaq; Promega, Madison, Wisconsin, USA). Primers Myco-f 5′- GAA GCT TAT GGT ACA GGT TGG -3′ and Myco-r 5-ATT ACC ATC CTT GTT GTA AGG -3′, Clam-1f-5′- TGC ATA ACC TAC GGT GTG TT -3′ and Clam-1r 5′- TGC ATA ACC TAC GGT GTG TT -3′, Clam-2f-5′- AGT TGA GCA TAT TCG TGA TT -3′ and Clam-2r 5′- TTT ATT CCG TGT CGT CCA G -3′. Amplifications consisted of initial incubation at 95°C for 2 min, followed by 40 cycles of 95°C for 30 s; 58°C for 30 s, and 72°C for 30 s; with a final extension at 72°C for 5 min. Amplicons were detected as 275 and 225 for Mycoplasma pneumoniae and Chlamydia pneumoniae respectively base pair bands after gel electrophoresis and nucleic acid staining (SybrGreen, Promega).

In each PCR assay, negative (viral transport medium) and positive control (cDNA viral) were prepared with the same procedure. Amplified products were recovered from the gel, purified (SpinPrep Gel DNA Kit; San Diego, CA) and sent for commercial sequencing (Macrogen, Korea).

### Statistical analysis

Quantitative variables were described as frequencies and percentages for each group. Statistical Analysis including Chi square and Fisher’s exact test were performed using the GraphPad Prism3 statistical (Graph Pad Sofware Inc., San Diego, USA).

## Results

A total of 675 children under 18 years diagnosed with an acute respiratory infection admitted to the *“Hospital Nacional Cayetano Heredia*. *Lima—Peru”* were consecutively studied from May 2009 to September 2010. *Mycoplasma pneumoniae* was detected in 25.19% (170/675) of nasopharyngeal samples and *Chlamydia pneumoniae* in 10.52% (71/675). *M*. *pneumoniae* was more frequently reported in males 58.82% (100/170); on the contrary, for *C*. *pneumonia*e prevalence a similar sex distribution was observed “Table 1”.

**Table 1:** Demographic characteristics of children with acute respiratory infections.

Demographic Characteristics	Total of patients n = 675 (%)	*Chlamydia pneumoniae* n = 71 (%)	*Mycoplasma pneumoniae* n = 170 (%)
**Sex**			
Female	279 (41.33)	35 (49.30)	70 (41.33)
Male	396 (58.67)	36 (50.70)	100 (58.82)
**Age**			
≤ 28 days	98 (14.52)	5 (7.04)	18 (10.59)
29 days—2 months	120 (17.78)	19 (26.76)	36 (21.18)
3 months—5months	83 (12.29)	11 (15.49)	20 (11.76)
6 months—11 months	118 (17.48)	13 (18.31)	33 (19.41)
1 year—5 years	183 (27.11)	18 (25.35)	47 (27.65)
6 years-10 years	40 (5.93)	1 (1.41)	6 (3.53)
11 years—17 years	24 (3.56)	3 (4.23)	6 (3.53)
Unknown	9 (1.33)	1 (1.41)	4 (2.35)

*M*. *pneumoniae* was more commonly detected in children between 1 to 5 years old in 27.65% (47/170), followed by the infants with 29 days to 2 months old in 21.18% (36/170). In a similar fashion, for *C*. *pneumoniae*, the most common age group affected were infants with 29 days to 2 months old followed by children between 1 to 5 years old in 26.76% (19/71) and 25.35% (18/71) respectively “Table 1”.

The most common symptoms in patients with these atypical pathogens were rhinorrhea, cough and fever. For patients with samples positive for *M*. *pneumoniae*, both cough and rhinorrhea were the most frequent found in 81.76% (139/170) and fever in 80.59% (137/170) cases; however only Rhinorrhea was statistical significant for this group (p = 0.014). In patients with *C*. *pneumoniae*, rhinorrhea was also statistically significant (p = 0.01) and the most predominant symptom found in 87.32% (62/71), followed by cough in 77.46% (55/71) and fever in 69.01% (49/71) “Table 2”. Moreover, during the hospitalization, community acquired pneumonia (CAP) was the most common diagnosis for both groups. CAP was diagnosed in 37.65% (64/170) of patients positive for M. pneumoniae (p<0.001) and in 23.94% (17/71) of patients with *C*. *pneumoniae* (p = 0.359). It is important to mention that for patients positive for *C*. *pneumoniae*, acute bronchial obstruction was the second most frequent presentation, observed in 12.68% (9/71) of cases (p = 0.03) followed by Asthmatic crisis in 9.86% (p = 0.001) “Table 3”.

**Table 2:** Signs and symptoms.

Clinical Symptoms	Total of Patients n = 675 (%)	*Chlamydia pneumoniae*		*Mycoplasma pneumoniae*	
		n = 71 (%)	p-value*	n = 170 (%)	p-value*
Fever	508 (75.26)	49 (69.01)	0.197	137 (80.59)	0.063
Cough	518 (76.74)	55 (77.46)	0.879	139 (81.76)	0.073
Throat pain	97 (14.37)	12 (16.90)	0.520	25 (14.71)	0.885
Rhinorrhea	504 (74.66)	62 (87.32)	0.010	139 (81.76)	0.014
Sputum	180 (26.66)	12 (16.90)	0.049	46 (27.06)	0.894
Respiratory wheezing	271 (40.14)	27 (38.03)	0.700	76 (44.71)	0.161
Pharyngeal congestion	185 (0.27)	25 (35.21)	0.119	48 (28.24)	0.780
Otalgia	9 (1.33)	1 (1.41)	0.953	1 (0.59)	0.462
Photophobia	4 (0.59)	1 (1.41)	0.344	2 (1.18)	0.265
Conjunctival Congestion	31 (4.59)	6 (8.45)	0.101	4 (2.35)	0.107
Vomits	93 (13.78)	13 (18.31)	0.241	31 (18.24)	0.051
Abdominal Pain	28 (4.15)	6 (8.45)	0.055	6 (3.53)	0.640
Diarrhea	74 (10.96)	12 (16.90)	0.090	25 (14.71)	0.071
Lymphadenopathies	17 (2.52)	4 (5.63)	0.093	5 (2.94)	0.777
Asthenia	66 (0.10)	10 (14.08)	0.196	18 (10.59)	0.680
Headache	33 (4.89)	4 (5.63)	0.769	12 (7.06)	0.129
Myalgias	10 (1.48)	2 (2.82)	0.284	1 (0.59)	0.465
Malaise	179 (26.52)	23 (32.39)	0.256	45 (26.47)	0.988
Dermic lesions	7 (1.04)	0 (0.00)	1.000	1 (0.59)	0.687

*Pearson's Chi-square (χ^2^) / Fisher's exact statistic.

**Table 3:** Clinical symptoms in PCR confirmed cases for *Chlamydia pneumoniae* and *Chlamydia pneumoniae*.

Clinical Diagnosis	Total of Patients n = 675 (%)	*Chlamydia pneumoniae*		*Mycoplasma pneumoniae*	
		n = 71 (%)	p-value*	n = 170 (%)	p-value*
Influenza	53 (7.85)	3 (4.23)	0.230	10 (5.88)	0.039
Rhino pharyngitis	34 (5.03)	5 (7.04)	0.389	0 (0.00)	0.040
Pharyngitis	4 (0.59)	3 (4.23)	0.004	0 (0.00)	1.000
Common Cold	5 (0.74)	0 (0.00)	1.000	1 (0.59)	0.427
CROUP	1 (0.15)	0 (0.00)	1.000	1 (0.59)	0.105
Pneumonia	193 (28.6)	17 (23.94)	0.359	64 (37.65)	< 0.001
Acute bronchial obstruction	41 (6.07)	9 (12.68)	0.030	16 (9.41)	< 0.001
Bronchiolitis	56 (8.30)	7 (9.86)	0.614	17 (10.00)	< 0.001
Asthmatic Crisis	18 (2.67)	7 (9.86)	0.001	6 (3.53)	0.007
Seizures	4 (0.59)	1 (1.41)	0.360	1 (0.59)	0.360
Acute diarrhea	11 (1.63)	4 (5.63)	0.021	4 (2.35)	0.021
Encephalitis	7 (1.04)	0 (0.00)	1.000	2 (1.18)	0.162
Herpes	1 (0.15)	0 (0.00)	1.000	1 (0.59)	0.105
Respiratory infection	21 (3.11)	4 (5.63)	0.264	3 (1.76)	0.476
Meningitis	1 (0.15)	1 (1.41)	0.105	0 (0.00)	1.000
Meningoencephalitis	5 (0.74)	2 (2.82)	0.088	0 (0.00)	1.000
Paralysis	2 (0.30)	0 (0.00)	1.000	1 (0.59)	0.199
Sepsis	25 (3.70)	2 (2.82)	1.000	6 (3.53)	0.038
Whooping	9 (1.33)	0 (0.00)	0.608	5 (2.94)	0.001
Acute respiratory distress	6 (0.89)	2 (2.82)	0.124	2 (1.18)	0.124
Febrile syndrome	32 (4.74)	2 (2.82)	0.564	4 (2.35)	0.765
Guillain Barre Syndrome	1 (0.15)	0 (0.00)	1.000	1 (0.59)	0.105

*Pearson's Chi-square (χ^2^) / Fisher's exact statistic.

**Fig 1:**
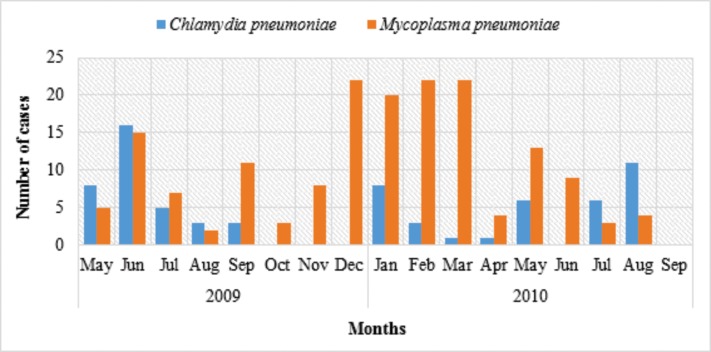
*C*. *pneumoniae* and *M*. *pneumoniae*. seasonal prevalence (May 2009 –Sep 2010).

A higher prevalence of *M*. *pneumoniae* cases were registered in summer, between December 2009 and March 2010. However, for C. *pneumoniae* no seasonal preference was observed. In 2009 a higher number of cases were reported between May to June and in 2010 most cases occurred in January and August Fig 1.

## Discussion

*Mycoplasma pneumoniae* and *Chlamydia pneumoniae* are known to be atypical pathogen responsible for pneumonia [5]. In the last years, there is an increasing interest to understand these pathogens, since both have been identified as important causes of morbidity and mortality in children [6,7,15,16].

*M*. *pneumoniae pneumonia* (MPP) occurs worldwide, and accounts for 10–40% of all cases of community-acquired pneumonia [6,10,17,18]. The prevalence of *C*. *pneumoniae* in children with ARI varies from 0 to 44% [3,18,19]. However, the information regarding the epidemiology of both bacterias in Peru is still limited. In our study, *Mycoplasma pneumonia* was detected in 25.19% of nasopharyngeal samples and *Chlamydia pneumonia* in 10.52%. One study in Peruvian children under 14 years old, reported a prevalence of *Chlamydia pneumoniae* 17.1% and *Mycoplasma pneumoniae* in 7.1%. Although, this study conducted in 2008 is one of the few available, they only included children hospitalized due to community-acquired pneumonia and IgM ELISA serology was performed for the detection of both bacterias [14]. In 2012, the Peruvian epidemiology department reported an increase of C. pneumoniae cases, recognizing this pathogen as the most frequent cause atypical pneumonia followed by *Mycoplasma pneumoniae*, affecting especially vulnerable population such as children and elderly [20]. Probably, because we used PCR as a diagnostic method and patients under 18 years old were included in our study a higher prevalence was obtained. Moreover, regarding *C*. *pneumoniae* infections, the 10.52% rate in children with ARIs found in the present study is slightly higher than the 6.7% found in a study performed by Kurz et al. in Vienna, Austria, and similar to the 9.3% reported by Schmidt et al. for Greifswald, Germany [21,22].

*M*. *pneumoniae* can be found in all age groups, with higher prevalence in children aged 5–15 years old [1,15,23]. Studies conducted in various countries have shown that Community-acquired *M*. *pneumoniae* or *C*. *pneumoniae* infections affect mainly preschool, school-aged children and young adults. Although, few studies have reported the frequency of *M*. *pneumoniae* and *C*. *pneumoniae* infections in infants [6]. In our study, both *M*. *pneumoniae* and *C*. *pneumoniae* were more prevalent in the infants with 29 days to 2 months old and children between 1 to 5 years old “Table 2”. The high prevalence of these bacterias in infants is especially worrisome since a previous study reported that the mean ages for severe *M*. *pneumoniae* CAP was around 21 months, about 49 months for severe *Chlamydia pneumoniae* CAP, and again about 24 months in severe co-infection pneumonia [24].

Bacterial and viral coinfections in children with *Mycoplasma pneumoniae pneumonia* (MPP) are a major issue that should be further studied; since co-infections are more common in severe MPP, and they also tend to be more serious increasing morbidity and hospitalization expenses [25,26]. Furthermore, *Chlamydia pneumonia* have been reported as one of the most frequent co-infected pathogens in children with MPP and can be detected in up to 25% of cases [27]. We found samples positive for both *M*. *pneumoniae* and *C*. *pneumoniae* in 25 (3.75%) cases and most of these patients were children under 6 month old, which is especially worrisome since they are a vulnerable population associated with more severe pneumonias and an increase risk of death [25,26].

*Mycoplasma pneumoniae* and *Chlamydia pneumoniae* are difficult to propagate, and can cause clinically indistinguishable disease patterns that can range from mild upper respiratory infections to sever atypical pneumonia [6–8]. As expected, in our study community acquired pneumonia (CAP) was the most common diagnosis for both bacterias and it was diagnosed in 37.65% of patients positive for *M*. *pneumoniae* (p<0.001) and in 23.94% of patients with *C*. *pneumoniae* (p = 0.359).

*Mycoplasma pneumoniae* is known to produce a gradual tracheobronchitis with malaise and nonproductive cough, which can progress to pneumonia and extra pulmonary manifestations [7,11]. In our study, both cough and rhinorrhea were the most common symptoms observed in children with *M*. *pneumoniae*, both found in 81.76%, followed by fever in 80.59% cases. *C*. *pneumoniae* can cause pharyngitis, sinusitis, bronchitis and pneumonia and a variable clinical presentation have been also described [6,8]. For the *C*. *pneumoniae* group, we also found rhinorrhea 87.32%, cough 77.46% and fever 69.01% to be the most frequent symptoms to be reported. However, only rhinorrhea was statistically significant for both: M. pneumoniae (p = 0.014) and C. pneumoniae (p = 0.01). A high prevalence of fever has been reported in our series, probably because we included only hospitalized children which may have a most severe clinical presentation. Moreover, children co-infected with *M*. *pneumoniae* and *C*. *pneumoniae* are found to have fever more frequently which tend to be more severe in children with more than 3 years old [6,7].

The role of *C*. *pneumoniae* in chronic respiratory illness and exacerbations of have been also studied [8] and appears to be involved more with asthma persistence than exacerbations [6]. In our study, we highlight the fact that, acute bronchial obstruction was the second most frequent diagnosis, observed in 12.68% of children with positive samples for *C*. *pneumoniae*. Moreover, both Acute bronchial obstruction (p = 0.03) and Asthmatic crisis (p = 0.001) were statistically significant for this group of patients.

It has been suggested that in settings where *M*. *pneumoniae* is endemic, seasonality may not be a factor, but when epidemics occur more cases are registered in the summer or early autumn, with no obvious explanation for this seasonal variation [6,28–30]. However, for *C*. *pneumoniae* no clear seasonality or correlation with climatic conditions have been stablished [6]. In our series, a higher prevalence of *Mycoplasma pneumoniae* cases were registered in summer, between December 2009 and March 2010. On the other hand, *Chlamydia pneumoniae* cases were higher in between May to June and in 2010 most cases occurred in January and August with no special seasonal preference.

Our study presented two limitations. First, the study was designed only for Mycoplasma pneumoniae and Chlamydia pneumoniae detection in the patient’s samples. Therefore, the presence of other common etiologies cannot be excluded. Furthermore, because other organisms that would cause similar symptoms were not tested we cannot establish causality between the clinical presentation in these patients and the atypical bacteria found in their samples. Another limitation was that physicians had no restrictions during the registration of their patients impressions, which caused a variety of clinical diagnosis aside from ARI, and we ignore the diagnostic criteria used by them. However, we want to highlight the fact that in our setting, patients hospitalized with different diagnosis can still be infected by *M*. *pneumoniae* and *C*. *pneumoniae*.

The study was a branch of a major investigation focus on the PCR detection of ARI etiologies including respiratory viruses and *Bordetella pertussis* in children under 18 years old from Lima, Peru [31,32]. After samples were obtained and stored, due to financial limitation and equipment availability, they were processed at different periods of time for different studies etiologies. However, due to the lack of data available in Peru about the prevalence of *M*. *pneumoniae* and *C*. *pneumoniae* in children we believe our results are still related to the current epidemiology in our population, since a similar prevalence of ARI have been observed in Peru in the last 7 years [13]. Our results demonstrate a considerable prevalence of both atypical pathogens in children previously diagnosed as acute respiratory infections and highlights the importance of their laboratory detection for proper and prompt antibiotic treatment.

In conclusion, *M*. *pneumoniae* and *C*. *pneumoniae* are a major health problem and it is necessary to monitor this atypical pneumonia causative bacteria in Peru. In the past, limitations in the diagnosis have impeded our ability to understand the epidemiology of the local outbreak setting as well as the spread of this pathogen. Recently, the detection of *M*. *pneumoniae* and *C*. *pneumoniae* in throat swab specimens by PCR has been found to be a highly sensitive and specific diagnostic technique for the diagnosis [6,11]. A national surveillance program for atypical pneumonia etiologies should be established in Peru, and further studies should evaluate the use of PCR as a reliable diagnostic method.
